# The association of gut microbiota characteristics in Malawian infants with growth and inflammation

**DOI:** 10.1038/s41598-019-49274-y

**Published:** 2019-09-09

**Authors:** Arox W. Kamng’ona, Rebecca Young, Charles D. Arnold, Emma Kortekangas, Noel Patson, Josh M. Jorgensen, Elizabeth L. Prado, David Chaima, Chikondi Malamba, Ulla Ashorn, Yue-Mei Fan, Yin B. Cheung, Per Ashorn, Kenneth Maleta, Kathryn G. Dewey

**Affiliations:** 1 Department of Biomedical Sciences, University of Malawi, College of Medicine, Blantyre, Malawi; 2 Program in International and Community Nutrition and Department of Nutrition, University of California, Davis, CA USA; 3 Center for Child Health Research, Faculty of Medicine and Health Technology, University of Tampere, Tampere, Finland; 4 School of Public Health and Family Medicine, University of Malawi, College of Medicine, Blantyre, Malawi; 5 Center for Quantitative Medicine, Duke-NUS Graduate Medical School, Singapore, Singapore; 6 Department of Paediatrics, Tampere University Hospital, Tampere, Finland

**Keywords:** Metagenomics, Paediatric research

## Abstract

We tested the hypotheses that a more mature or diverse gut microbiota will be positively associated with infant growth and inversely associated with inflammation. We characterized gut microbiota from the stool samples of Malawian infants at 6 mo (n = 527), 12 mo (n = 632) and 18 mo (n = 629) of age. Microbiota diversity and maturity measurements were based on Shannon diversity index and microbiota for age Z-score (MAZ), respectively. Growth was calculated as change in Z-scores for weight-for-age (WAZ), length-for-age (LAZ) and head circumference-for-age (HCZ) from 6 to 12 mo and 12 to 18 mo. Biomarkers of inflammation (alpha-1-acid glycoprotein (AGP) and C-reactive protein (CRP)) were measured at 6 and 18 mo. Multivariable models were used to assess the association of each independent variable with each outcome. Microbiota diversity and maturity were related to growth in weight from 6 to 12 mo, but not to growth in length or head circumference or to growth from 12 to 18 mo. Microbiota diversity and maturity may also be linked to inflammation, but findings were inconsistent.

## Introduction

The human gut is colonized by a vast array of microorganisms, which are largely commensal^1^. These microorganisms constitute what is known as the gut microbiota, and they play an important role in the development of the host immune system^2^ and many physiological functions that are important for the survival of the host^3^. Alterations to the normal gut microbial status (dysbiosis) have been associated with obesity^4^, kwashiorkor^5^ and inflammatory diseases^6–8^. It has been reported that inflammatory conditions such as Crohn’s disease (CD) and ulcerative colitis (UC) are related to the loss of enteric bacterial diversity^9,10^. A recent study in animal models demonstrated that enrichment of Enterobacteriaceae was associated with the development of CD, while depletion of this bacterial family led to a reduction in inflammation^11^. It has also been shown that a depletion of *Faecalibacterium prausnitzii* was associated with the recurrence of CD in mice with chemically induced colitis, while supplementing the mice with this bacterium led to a reduction in inflammation^12^. In healthy children, it is reported that there is rapid and high diversification of bacterial microbiome over the first year of life, however this diversification is delayed and lower in children with allergy and asthma^13,14^ or in those who are malnourished^15^. A study in Malawian children demonstrated an association of specific bacterial taxa with environmental enteric dysfunction (EED), a chronic condition of intestinal inflammation and blunting of intestinal villi^16^.

Experimental work in gnotobiotic mice implanted with stool samples from Malawian infants has shown that gut microbiota composition and maturity are associated with growth status^5,15,17^. Furthermore, undernourished children exhibited an immature microbiota, which transmitted impaired growth phenotypes in mice models^17^. In the sample of children from the latter study, microbiota maturity was positively associated with anthropometric status at 18 mo of age^17^, but the analysis was confined to correlations with attained growth status and did not examine change in growth status over time. A study in the Gambia reported associations of the gut microbiota with infant morbidity, inflammation and growth^18^, but the sample size (n = 33) was too small to permit definitive conclusions. Another study reported that linear growth faltering was associated with the presence of Acidaminococcus and community-level changes in the gut microbiota^19^.

While it is clear from these reports that the gut microbiota composition plays an influential role in inflammation and enteropathy^16,20^, which may be linked to growth faltering^21,22^, the nature of this relationship and the functional consequences of variations in the gut microbiota during infancy remain to be fully understood.

We used prospective data from a large cohort of children in Malawi to investigate whether characteristics of the microbiota in infancy are associated with growth and inflammation. We tested the following hypotheses: (i) a more mature or diverse microbiota at 6 or 12 mo will be positively associated with infant growth during the subsequent six months, based on change in length for age z-score (LAZ), weight for age z-score (WAZ), weight for length z-score (WLZ), and head circumference z-score (HCZ), (ii) inverse relationships will be observed between a more mature or diverse microbiota and concurrent biomarkers of inflammation at 6 mo and 18 mo, and between a more mature or diverse microbiota at 6 mo or 12 mo and future inflammation at 18 mo. We also investigated, as a secondary objective, the association of specific bacterial taxa with infant growth, based on change in LAZ, WAZ, and WLZ.

## Results

### Study profile and follow-up outcome

**Figure 1:**
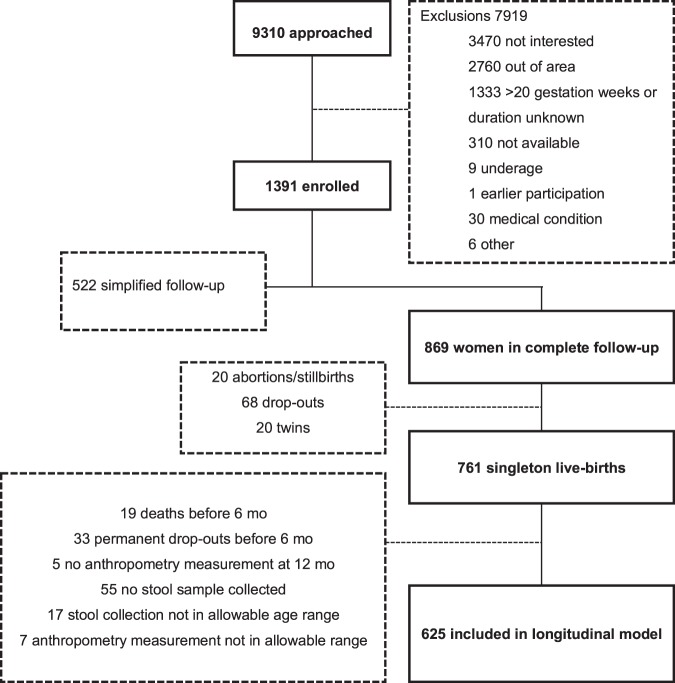
Study profile and follow-up.

Among the 869 mothers assigned to the follow-up study (Fig. 1), 761 singleton live births were reported. The women who were not included experienced either spontaneous abortions/stillbirths (n = 20), dropped out of the study (n = 68) or gave birth to twins (n = 20). At 18 mo, 622 children completed anthropometric measurements and the rest (n = 138) were lost to follow-up. Data on microbiota composition of stool samples were available for 515 children at 6 mo and 630 children at 12 mo. The increase in the number of children from 6 to 12 mo was due to the higher prevalence of diarrhoea cases at 6 mo which prevented stool collection.

### Baseline characteristics and infant gut microbiota characteristics

At baseline, the mothers excluded from this sub study were similar to the mothers included in the study for most of the characteristics considered (Table 1). However, those excluded were from households with a higher BMI, higher mean asset score and lower likelihood of severe food insecurity. The mean (SD) of MAZ was 0.64 (2.92) at 6 mo, −0.28 (2.66) at 12 mo, and −1.32 (1.76) at 18 mo. The decrease in MAZ score with age suggests a worsening relative microbiota maturity in this cohort, which parallels the worsening of height-for-age Z-scores in the same setting. The mean (SD) of MAZ, at all time-points in the longitudinal model, was 0.08 (2.48). The mean (SD) of Shannon index was 1.61 (0.65) at 6 mo, 2.40 (0.67) at 12 mo, and 2.94 (0.62) at 18 mo. The mean (SD) of Shannon index, at all time-points in the longitudinal model, was 2.03 (0.73). Shannon index and MAZ were substantially correlated at each time-point. The Spearman’s correlation coefficients were 0.65 at 6 mo, 0.76 at 12 mo, and 0.76 at 18 mo. The Spearman’s correlation coefficient for the longitudinal association was 0.49.

**Table 1:** Characteristics of included and excluded participants.

Characteristic	Included	Excluded	*p*-value
Participants, n	691	707	
Maternal age at enrollment, years	25.2 (5.9)	24.8 (6.2)	0.20
Maternal height, cm	156.1 (5.7)	156.0 (5.6)	0.68
Maternal BMI, kg/m^2^	22.0 (2.8)	22.4 (2.9)	0.04
Maternal education completed, years	3.8 (3.5)	4.2 (3.4)	0.08
Positive malaria RDT of the mother at enrollment	22.4%	23.9%	0.52
HIV+ status of the mother	11.9%	15.5%	0.06
Mode of delivery (% with vaginal delivery)	94.9%	93.4%	0.28
Food insecure household	38.9%	33.1%	0.03
Access to sanitary facility (% with flash toilet)	9.6%	8.5%	0.57
Household asset Z-score	−0.08 (1.0)	0.09 (1.0)	0.01
LAZ at 6 mo	−1.3 (1.1)	−1.1 (1.1)	0.08
WAZ at 6 mo	−0.57 (1.2)	−0.56 (1.1)	0.89
High AGP at 6 mo	64.4%	—	
High CRP at 6 mo	28.8%	—	

Values are in mean (standard deviation) or percentages. *p*-values are obtained from t-test (continuous variables) or chi-square test (proportions). BMI (Body mass index). RDT (rapid diagnostic test). LAZ (Length-for-age Z-score). WAZ (Weight-for-age Z score). AGP (Alpha-1 acid glycoprotein). C-reactive protein (CRP).

### Association of microbiota maturity or diversity with infant growth from 6–12 or 12–18 mo

**Figure 2:**
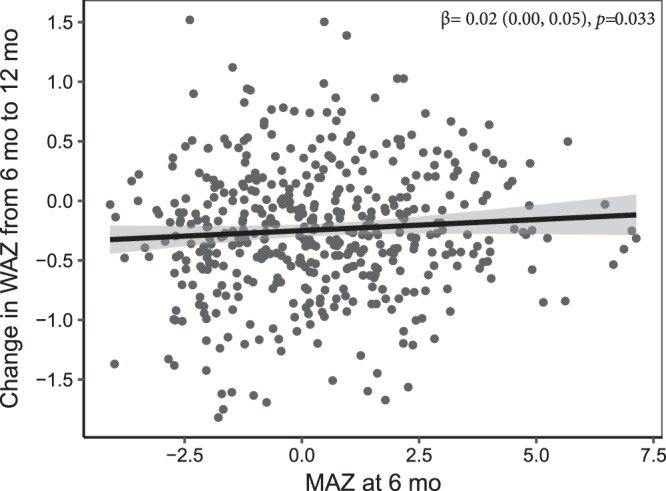
Association of MAZ at 6 mo of age with change in WAZ from 6 to 12 mo of age. The figure was generated using data from adjusted cross-sectional models and shows a positive relationship between microbiota maturity (MAZ) and change in WAZ from 6 to 12 mo (*p* = 0.033). The positive relationship was also observed in un-adjusted models (*p* = 0.001).

There was no significant interaction between MAZ and time regarding change in LAZ, HCZ or WLZ in either unadjusted or adjusted models (Table 2). MAZ was negatively associated with change in HCZ (*p* < 0.0001) and positively associated with change in WLZ (*p* < 0.0001) from 6 to 18 mo in unadjusted models, but these associations became non-significant in adjusted models. There was an interaction between MAZ and time regarding change in WAZ; accordingly, cross-sectional models were examined, which revealed that MAZ at 6 mo was positively related to change in WAZ from 6 to 12 mo in both unadjusted and adjusted models (Table 3 and Fig. 2), whereas there was no significant association of MAZ at 12 mo with change in WAZ from 12 to 18 mo.

**Table 2:** The longitudinal association of microbiota maturity at 6 or 12 mo with infant growth from 6 to 18 mo of age.

Microbiota maturity (MAZ)							
Outcome (n)	Unadjusted			Outcome (n)	Adjusted		
	β of MAZ in model without interaction	*p*-value			β of MAZ in model without interaction	*p*-value	
		Interaction, age-interval * MAZ	β of MAZ			Interaction, age-interval * MAZ	β of MAZ
LAZ (1058)	−0.01 (−0.02, 0.01)	0.308	0.364	LAZ (993)	−0.01 (−0.02, 0.01)	0.393	0.371
HCZ (1052)	−0.18 (−0.22, −0.14)	0.101	<0.0001	HCZ (998)	−0.01 (−0.02, 0.00)	0.199	0.171
WLZ (1059)	0.01 (0.00, 0.03)	0.125	<0.0001	WLZ (992)	0.01 (−0.01, 0.03)	0.159	0.37
WAZ (1057)		0.019		WAZ (999)		0.039	

The relationship between microbiota maturity (MAZ) and each pre-defined outcome (LAZ, length-for-age *z* score; HCZ, head-circumference *z* score; WLZ, weight-for-length *z* score; and WAZ, weight-for-age z score) was tested for significance in multivariate models while controlling for other factors, including nutrition intervention group. The β of MAZ in the model without the interaction term was derived from a model including terms for the age interval and MAZ, but no interaction of age-interval and MAZ. The term ‘Age-interval *MAZ’ tested the significance of the interaction between time and microbiota maturity in a longitudinal model. The repeated measures models included two age intervals based on child’s age; in the first age interval, the response variable was change in z-score between 6 and 12 mo and the predictor was MAZ at 6 mo, and in the second age interval the response was change in z-score between 12 and 18 mo and the predictor was MAZ measured at 12 mo. The *p*-values were obtained from repeated measures ANCOVA. The models were adjusted for the following pre-specified covariates: intervention group; child age on day of stool collection; maternal age, height, body mass index, parity, education, HIV status, and hemoglobin at enrollment; household assets, food security, source of drinking water (tap water vs any other source), residential location and access to sanitary facility (water closet or ventilation improved pit latrine vs. none or regular pit latrine); season at time of stool sample collection; mode of delivery (vaginal or cesarean); site of delivery; and child sex.

**Table 3:** Association of microbiota maturity with growth in weight when the interaction between age interval and MAZ was significant.

Outcome	MAZ time point of measurement (n)	Unadjusted		Adjusted	
		β of MAZ	*p* of β of MAZ	β of MAZ	*p* of β of MAZ
ΔWAZ (6–12 mo)	6 mo (n = 468)	0.03 (0.01, 0.05)	0.001	0.02 (0.00, 0.05)	0.033
ΔWAZ (12–18 mo)	12 mo (n = 592)	−0.02 (−0.03, 0.01)	0.463	−0.01 (−0.03, 0.01)	0.35

The relationship between microbiota maturity (MAZ) and change in WAZ (weight-for-age z score) when there was a significant interaction between age interval and MAZ. The relationship was assessed for both unadjusted and adjusted models. The *p*-values were obtained from repeated measures ANCOVA. The models were adjusted for the following pre-specified covariates: intervention group; child age on day of stool collection; maternal age, height, body mass index, parity, education, HIV status, and hemoglobin at enrollment; household assets, food security, source of drinking water (tap water vs any other source), residential location and access to sanitary facility (water closet or ventilation improved pit latrine vs. none or regular pit latrine); season at time of stool sample collection; mode of delivery (vaginal or cesarean); site of delivery; and child sex.

**Figure 3:**
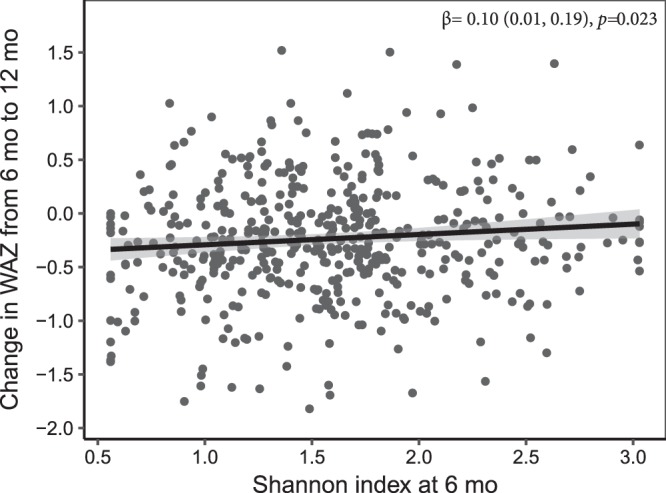
Association between microbiota diversity at 6 months of age and change in WAZ from 6 to 12 mo of age. The figure was generated using data from adjusted cross-sectional models and shows a positive relationship of Shannon index with change in WAZ from 6 to 12 mo (*p* = 0.023). The positive relationship was also observed in un-adjusted models (*p* = 0.002).

The interaction between Shannon index and time was not significant for change in LAZ or HCZ in either unadjusted or adjusted models (Table 4). Diversity was not associated with change in LAZ or HCZ from 6 to 18 mo in unadjusted or adjusted models. There was an interaction between Shannon index and time regarding change in WAZ and WLZ (although the latter became marginally significant in the adjusted model) (Table 4); accordingly, cross-sectional models were examined, which revealed that diversity was positively related to change in WAZ (Table 5 and Fig. 3) and WLZ (Table 5) from 6 to 12 mo in both unadjusted and adjusted models, whereas there was no significant association with change in WAZ or WLZ from 12 to 18 mo.

**Table 4:** The longitudinal association of Shannon diversity index at 6 or 12 mo with infant growth from 6 to 18 mo of age.

Outcome (n)	Microbiota diversity (Shannon index)					
	Unadjusted			Adjusted		
	β of Shannon index in model without interaction	*p*-value		β of Shannon index in model without interaction	*p*-value	
		Interaction, age-interval * Shannon index	β of Shannon index		Interaction, age-interval * Shannon index	β of Shannon index
LAZ (1058)	−0.02 (−0.08, 0.04)	0.365	0.539	−0.03 (−0.09, 0.04)	0.485	0.397
HCZ (1052)	−0.01 (−0.06, 0.05)	0.158	0.838	−0.01 (−0.06, 0.05)	0.338	0.847
WLZ (1059)		0.025		0.03 (−0.04, 0.10)	0.054	0.436
WAZ (1057)		0.006			0.037	

The relationship between microbiota diversity (Shannon index) and growth (LAZ, length-for-age *z* score; HCZ, head-circumference *z* score; and WLZ, weight-for-length *z* score) was tested for significance in multivariate models while controlling for other factors, including nutrition intervention group. The β of Shannon index in the model without the interaction term was derived from a model including terms for the age interval and Shannon index, but no interaction of age-interval and Shannon index. The term ‘Age-interval *H’ tested the significance of the interaction between age interval and microbiota diversity in a longitudinal model. The repeated measures models included two age intervals based on child’s age; in the first age interval, the response variable was change in z-score between 6 and 12 mo and the predictor was Shannon index at 6 mo, and in the second age interval, the response was change in z-score between 12 and 18 mo and the predictor was Shannon index measured at 12 mo. The *p*-values were obtained from repeated measures ANCOVA. The models were adjusted for the following pre-specified covariates: intervention group; child age on day of stool collection; maternal age, height, body mass index, parity, education, HIV status, and hemoglobin at enrollment; household assets, food security, source of drinking water (tap water vs any other source), residential location and access to sanitary facility (water closet or ventilation improved pit latrine vs. none or regular pit latrine); season at time of stool sample collection; mode of delivery (vaginal or cesarean); site of delivery; and child sex.

**Table 5:** Association of microbiota diversity with growth in weight when the interaction between age interval and Shannon index was significant.

Outcome	Shannon index time point of measurement	Unadjusted		Adjusted	
		β of Shannon index	*p* of β of Shannon index	β of Shannon index	*p* of β of Shannon index
ΔWAZ (6–12 mo)	6 mo	0.14 (0.05, 0.25)	0.002	0.10 (0.01, 0.19)	0.023
ΔWAZ (12–18 mo)	12 mo	−0.03 (−0.11, 0.05)	0.469	0.04 (−0.15, 0.05)	0.35
ΔWLZ (6–12 mo)	6 mo	0.16 (0.05, 0.28)	0.005	0.15 (0.04, 0.26)	0.01
ΔWLZ (12–18 mo)	12 mo	−0.01 (−0.10, 0.09)	0.879	−0.03 (−0.13, 0.07)	0.59

The relationship between microbiota diversity (Shannon index) and change in WAZ (weight-for-age z score) and WLZ (weight-for-length z score) when there was a significant interaction between age interval and Shannon index. The relationship was assessed for both unadjusted and adjusted models. The *p-*values were obtained from repeated measures ANCOVA. The models were adjusted for the following pre-specified covariates: intervention group; child age on day of stool collection; maternal age, height, body mass index, parity, education, HIV status, and hemoglobin at enrollment; household assets, food security, source of drinking water (tap water vs any other source), residential location and access to sanitary facility (water closet or ventilation improved pit latrine vs. none or regular pit latrine); season at time of stool sample collection; mode of delivery (vaginal or cesarean); site of delivery; and child sex.

### Association of microbiota maturity or diversity with biomarkers of inflammation at 6 and 18 mo of age

**Figure 4:**
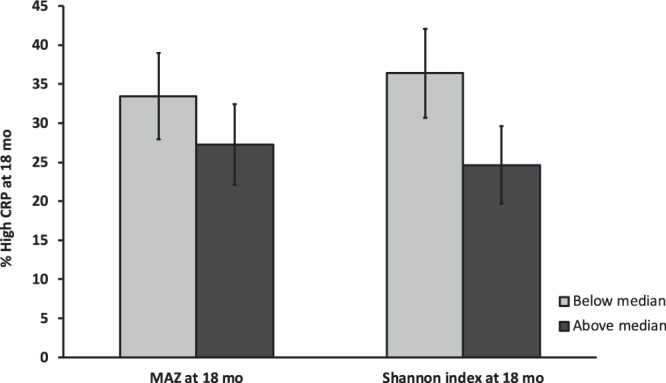
Association of inflammation with MAZ and microbiota diversity. The figure shows the concurrent association of acute inflammation with MAZ and Shannon index at 18 mo of age. The light grey bars show the percentage of high CRP for values of MAZ or Shannon index below the median. The dark grey bars show the percentage of high CRP for values of MAZ or Shannon index above the median. The percentage with high CRP is higher for values of MAZ (*p* = 0.111) or Shannon index (*p* < 0.001) below the median.

There was no predictive association between MAZ at 6 mo or 12 mo and biomarkers of inflammation at 18 mo (data not shown). MAZ at 6 mo was not associated with concurrent inflammation but MAZ at 18 mo was associated with inflammation at 18 mo based on CRP concentration and high CRP (Table 6). For a one Z-score unit increase in MAZ, there was a 12% decrease in CRP concentration (β = 0.88, 95% CI: 0.88 (0.80, 0.96), *p* = 0.003)) and the odds of high CRP decreased by 14% (OR:0.86, 95% CI: (0.78, 0.96), *p* = 0.009). There was a higher percentage of high CRP values at 18 mo for concurrent MAZ below median compared with MAZ above median (Fig. 4).

**Table 6:** The association of microbiota maturity (MAZ) or microbiota diversity (Shannon index) at 6 or 18 mo with inflammation.

Predictor Variable	Age for measured inflammation outcomes	Inflammation outcome variables							
		Alpha-1 acid glycoprotein (AGP)*		C-reactive protein (CRP)*		High AGP		High CRP	
		β- value (95% CI)	*p*-value	β-value (95% CI)	*p*-value	OR (95% CI)	*p*-value	OR (95% CI)	*p*-value
MAZ (6 mo)	6 mo	1.01 (0.99, 1.03)	0.17	0.97 (0.90, 1.06)	0.47	1.03 (0.90, 1.09)	0.849	1.09 (0.99, 1.19)	0.072
Shannon index (6 mo)		1.06 (1.02, 1.10)	0.042	0.92 (0.86, 1.89)	0.6	1.39 (0.97, 2.00)	0.072	1.04 (0.71, 1.54)	0.831
MAZ (6 mo)	18 mo	1.00 (0.98, 1.01)	0.780	0.99 (0.92, 1.06)	0.748	1.00 (0.91, 1.09)	0.943	1.03 (0.94, 1.13)	0.501
Shannon index (6 mo)		0.97 (0.91, 1.03)	0.375	0.90 (0.66, 1.22)	0.487	1.00 (0.71, 1.41)	0.992	1.12 (0.79, 1.60)	0.530
MAZ (18 mo)	18 mo	1.01 (0.98, 1.03)	0.89	0.88 (0.80, 0.96)	0.0034	0.97 (0.88, 1.09)	0.618	0.86 (0.78, 0.96)	0.009
Shannon index (18 mo)		0.97 (0.92, 1.03)	0.22	0.64 (0.55, 0.92)	0.01	0.85 (0.62, 1.16)	0.31	0.68 (0.50, 0.93)	0.016

Inflammation was assessed by measuring Alpha-1 acid glycoprotein (AGP) and C-reactive protein (CRP) from blood/plasma as biomarkers reported as g/L and mg/L respectively. High AGP was defined as [AGP] value >1.0 g/L and high CRP as [CRP] value >5.0 mg/L. The association of MAZ and Shannon index with inflammation was assessed by controlling for child age on day of stool collection; maternal age, height, body mass index, parity, education, HIV status, and hemoglobin at enrollment; household assets, food security, source of drinking water (tap water vs any other source), residential location and access to sanitary facility (water closet or ventilation improved pit latrine vs. none or regular pit latrine); season at time of stool sample collection; mode of delivery (vaginal or cesarean); site of delivery; and child sex. There was no difference in findings from the unadjusted and adjusted models, so this table only shows adjusted models. *Beta values are back-transformed from a natural log transformation.

Microbial diversity at 6 mo was associated with AGP at 6 mo but not CRP (Table 6): for a one-unit increase in Shannon index, there was a 5.9% increase in AGP concentration (g/L) (β = 1.06, 95% CI: (1.02, 1.10), *p* = 0.042). However, there was no association between microbiota diversity at 6 mo or 12 mo and biomarkers of inflammation at 18 mo (data not shown). Microbiota diversity at 18 mo was associated with CRP and high CRP at 18 mo, while no relationship was observed with AGP or high AGP. For a one-unit increase in Shannon index at 18 mo, there was a 36% decrease in CRP (β = 0.64, 95% CI: (0.55, 0.92), *p* = 0.01) concentration at 18 mo, and the odds of a high CRP decreased by 32% (OR = 0.68, 95% CI: (0.50, 0.93), *p* = 0.016). There was a higher percentage of high CRP values at 18 mo for concurrent Shannon index below median compared with Shannon index above median (*p* < 0.001) (Fig. 4).

### Taxa associated with infant growth

**Figure 5:**
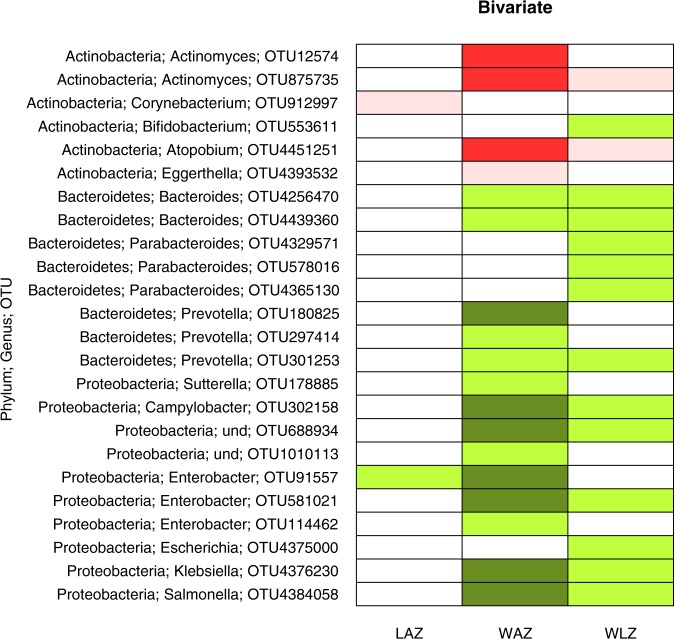
Taxa specific associations with growth. The figure shows negative and positive associations of specific taxa with changes in z-scores between 6 and 12 mo: LAZ, length-for-age z score; WAZ, weight-for-age z score; and WLZ, weight-for-length z score. The negative associations that were significant only before FDR correction (at 15%) and those that remained significant after correction are shown in pink and dark red, respectively. Positive associations that were significant only before correction are shown in light green colour, while those that remained significant after correction are in dark green colour.

**Figure 6:**
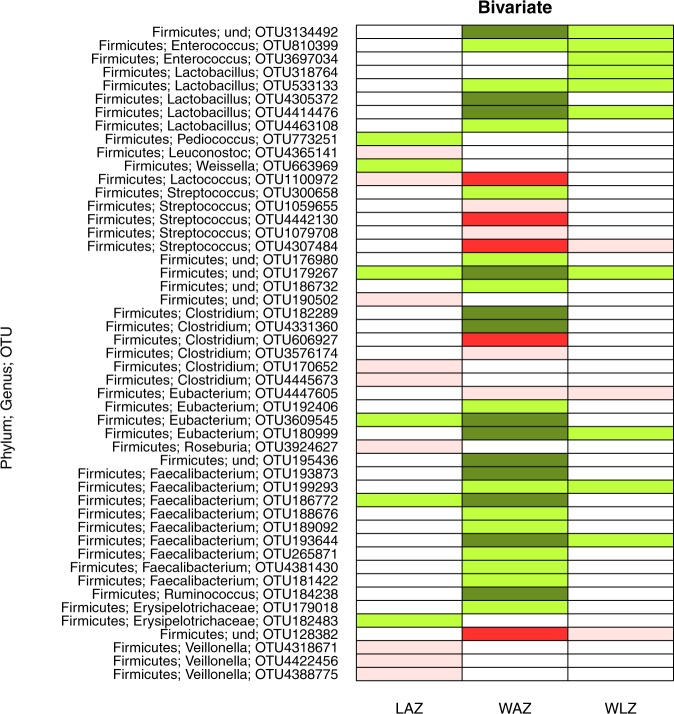
Taxa specific associations with growth, for Firmicutes phylum only. The figure shows negative and positive association of specific taxa changes in z-scores between 6 and 12 mo: LAZ, length-for-age z score; WAZ, weight-for-age z score; and WLZ, weight-for-length z score. The negative associations that were significant only before FDR correction at 15% and those that remained significant after correction are shown in pink and dark red, respectively. Positive associations that were significant only before correction are shown in light green color, while those that remained significant after correction are in dark green color.

Because we found that MAZ and Shannon index at 6 mo were related to infant growth from 6 to12 mo, we examined the specific taxa at 6 mo associated with growth for the 6–12 mo period. No further analysis of specific taxa at 12 mo was conducted since there was no significant association of either MAZ or Shannon index at 12 mo with change in Z scores from 12 to18 mo. At 6 mo, there were 7428 OTUs in total; following filtering steps, only 291 OTUs remained. Of these, 64% (187/291) and 60% (174/291) were associated with Shannon index and MAZ (*p* < 0.05), respectively. Only a few of the 291 OTUs were significantly associated with at least one of the growth outcomes (change in LAZ, WAZ or WLZ, Figs 5 and 6), either positively (shown in green) or negatively (shown in red). Those shown in light green or pink were significant only before the FDR correction; those in dark green or red remained significant after the FDR correction. Generally, there were more positive associations than negative associations. In addition, there were very few associations with linear growth (change in LAZ), whereas numerous significant associations with weight (change in WAZ or WLZ) were observed. The associations with change in WAZ and WLZ generally occurred in the same direction, i.e. if a given taxon was positively associated with change in WAZ, it was also positively associated with change in WLZ, with the same trend observed for negative associations.

Regarding the specific taxa at 6 mo associated with change in weight between 6 and 12 mo, we detected 8 OTUs that were negatively associated with change in WAZ. These included Actinomyces, *Actinomyces graevenitzii*, *Atopobium parvulum* (Fig. 5) and Lactococcus, Streptococcus, *Streptococcus mitis*, and *Clostridium difficile* (Fig. 6). We detected 20 OTUs that were positively associated with change in WAZ. These included Prevotella, Campylobacter, Enterobacteriaceae (family), *Enterobacter ludwigii*, Enterobacter_sp_A5_2, Klebsiella (genus), *Salmonella enterica* (Fig. 5) and Clostridium *Lactobacillus rogosae*, *Eubacterium hallii*, *Eubacterium rectale*, *Faecalibacterium prausnitzii*, and *Ruminococcus obeum* (Fig. 6). There were 5 OTUs that were negatively associated with change in WLZ and 23 OTUs positively associated with change in WLZ, however these associations did not remain significant after FDR correction. Overall, we observed that Proteobacteria and Bacteroidetes were positively associated with weight growth, while Actinobacteria (except for *Bifidobacterium dentium*) taxa were negatively associated with weight growth (Fig. 5).

Regarding the specific taxa associated with growth in length, we observed 10 and 7 OTUs (Figs 5 and 6) that were negatively and positively associated with change in LAZ, respectively. However, these associations did not remain significant following FDR adjustment.

### Taxa associated with inflammatory biomarkers

We examined taxa at 6 mo associated with AGP concentration at 6 mo because we observed a significant association of that outcome with microbiota diversity at 6 mo in the primary analyses. We detected 5 OTUs that were either negatively or positively related to AGP at 6 mo. We observed that when *Slackia isoflavoniconvertens* species was present at any abundance, AGP was lower compared to when the species was absent (β = −0.14 (−0.27, −0.01), *p* = 0.03). The presence of other taxa such as *Ruminococcus gnavus* (β = −0.16 (0.05, 0.27), *p* = 0.01), Lactobacillus (β = −0.26 (0.08, 0.45), *p* = 0.01), Clostridiales (β = −0.13 (0.04, 0.23), *p* = 0.01) and Clostridium (β = 0.1 (0.00, 0.20), *p* = 0.05) was positively associated with AGP at 6 mo. The relationship between *Slackia isoflavoniconvertens*, *Ruminococcus gnavus*, and Lactobacillus and AGP remained significant after FDR correction. To help understand these associations, we further examined the association of these taxa with Shannon index. We observed a positive and significant relationship of *Ruminococcus gnavus* (β = 0.22 (0.08, 0.35), *p* = 0.0018), *Slackia isoflavoniconvertens* (β = 0.61 (0.44, 0.78), *p* = 0.0001), Clostridiales (β = 0.27 (0.15, 0.39), *p* = 0.0001) and Clostridium (β = 0.32 (0.20, 0.45), *p* = 0.0001) with Shannon index at 6 mo. Each relationship remained significant after FDR correction. However, Enterobacteriaceae and Lactobacillus were not associated with Shannon index.

We examined taxa at 18 mo associated with CRP concentration at 18 mo because we observed significant associations of that outcome with both microbiota maturity and diversity at 18 mo. Several taxa exhibited a negative association with CRP at 18 mo including Prevotella (β = −1 (−1.9, −0.11), *p* = 0.03) and Leuconostoc (β = −0.87 (−1.59, −0.16), *p* = 0.02), whereas *Clostridium innocuum* (β = 0.89 (0.18, 1.6), *p* = 0.01) exhibited a positive association. The relationship between *Clostridium innocuum* and CRP at 18 months remained significant after FDR correction. Except for Leuconostoc, which was not related to Shannon index or MAZ, Prevotella and *Clostridium innocuum* were positively related to Shannon index (β = 0.29 (0.16, 0.41), p < 0.0001; β = 0.19 (0.03, 0.34), *p* = 0.02, respectively) and MAZ (β = 0.29 (0.16, 0.41) p < 0.0001; β = 0.19 (0.03, 0.34), *p* = 0.02, respectively) at 18 mo.

## Discussion

We investigated whether characteristics of the gut microbiota in infancy are associated with subsequent growth and inflammation. We first tested the hypothesis that a more mature or diverse microbiota will be positively associated with infant growth. There was no association between MAZ and growth from 6 to 18 mo in relation to LAZ, HCZ or WLZ. MAZ at 6 mo was positively, though weakly, related to change in WAZ from 6 to 12 mo, while MAZ at 12 mo was not related to change in WAZ from 12 to 18 mo. We observed no association between Shannon index and change in LAZ or HCZ from 6 to 18 mo. However, there was a positive relationship of Shannon index at 6 mo with change in WAZ and WLZ from 6 to 12 mo, whereas no association of Shannon index at 12 mo with change in WAZ or WLZ from 12 to 18 mo was observed. Next, we tested the hypothesis that a more mature or diverse microbiota will be inversely related to biomarkers of inflammation (CRP and AGP). At 6 mo, microbiota maturity was not related to either biomarker, but Shannon index was positively related to AGP concentration at the same time point. At 18 mo, both microbiota maturity and Shannon index were inversely related to CRP (but not AGP) concentration at the same time point.

The positive associations of both MAZ and Shannon index with growth in weight between 6 and 12 mo, but not between 12 and 18 mo, suggests that the second half of infancy is a critical period. Around 6 mo, the transition from predominant breastfeeding to a mixed diet with increasing amounts of complementary foods is underway, which has been associated with a sudden and major shift in the gut microbiome profile^23^ as demonstrated in piglets^24^. Variations in the microbiota profile at 12 mo may be less consequential for growth than those at earlier ages, given that complementary foods are well established in the diet by that age. In a cross-sectional study among children in Bangladesh at 18 mo of age, WLZ was not correlated with Shannon index but was inversely correlated with MAZ^15^. The lack of association of WLZ with microbial diversity is consistent with our findings during the 12–18 mo period, but the inverse association with MAZ is in conflict with our findings. The longitudinal nature of our study, and the inclusion of data on the gut microbiota during the first year of life, provide information that is not available from previous studies, which may help to explain these contradictory findings.

Regarding the specific taxa that might be related to growth from 6 to 12 mo, we found several associations that remained significant following FDR correction. Change in WAZ between 6 and 12 mo was positively associated with presence of several strains that are capable of hydrolysing cellulose such as Prevotella, Ruminococcus sp, Clostridium sp, Eubacterium sp and Bacteroides^25,26^. Prevotella is also thought to improve glucose metabolism through promotion of increased glycogen storage^27^. It is possible that the metabolic role played by Prevotella and other cellulolytic bacteria increases the availability of glucose which subsequently promotes weight growth. An unexpected finding was a significant positive association of *Salmonella enterica* with weight growth. *Salmonella enterica* is a foodborne pathogen responsible for inflammatory disease in the intestine following diarrhoea and is implicated in many deaths globally^28,29^. Taxa such as Actinobacteria (Actinomyces, Atopobium) and Firmicutes (Lactococcus, Streptococcus, and Clostridium) were negatively associated with change in WAZ. In contrast to our findings, a previous study in mice at weaning age showed that Firmicutes promoted weight gain, presumably because of the ability of these bacteria to digest complex sugars^30,31^; however, this could be driven by other genera and not necessarily Lactococcus, Streptococcus or Clostridium. In addition, metabolic effects in mice may not necessarily predict outcomes in humans. Although it has previously been reported that linear growth faltering is associated with taxa such as Acidaminococcus^19^, we did not find any significant association of any given taxa with linear growth in our study.

With regard to the associations between the microbiota and markers of inflammation, the finding of a concurrent positive relationship between Shannon index and AGP (a long-term biomarker of inflammation) at 6 mo and not CRP (a short-term biomarker of inflammation) was unexpected. Raised AGP levels (with normal CRP levels) are normally observed in subjects who have recovered from inflammatory conditions and are convalescing^32^. It is possible that inflammation at 6 mo may have been chronic (i.e pre-existing from early infancy) and thus that inflammation affected microbial composition rather than vice versa. At 18 mo however, a concurrent inverse relationship was observed between microbiota characteristics and CRP, though not AGP. Raised CRP levels are associated with recent infection with or without clinical evidence of disease^32^. The percentage of children with high CRP values was elevated among those with MAZ or Shannon index values below the median (compared with those above the median), by 6 and 12 percentage points, respectively. This suggests that a more mature and diverse microbiota community may help to prevent inflammation, but we cannot rule out the possibility that the relationship works in the opposite direction, i.e., that inflammation affects microbial diversity. Previous studies in adults demonstrated that lower alpha diversity and gene count of the gut microbiome were associated with higher levels of high sensitivity CRP^4,33^. High sensitivity CRP is an inflammatory biomarker for myocardial infarction, stroke and peripheral arterial diseases^34^. Mucosal inflammation in inflammatory bowel disease (IBD) has been associated with a significant reduction in the diversity of the gut microbiota measured by Shannon index^35–37^. Although determining whether variations in microbiota composition contribute to inflammation is a challenge in humans, it has recently been demonstrated in mice that a reduction in airway microbiota diversity was associated with elevated allergic respiratory inflammation^38^, suggesting that an altered microbiota profile can affect inflammation.

At 6 mo, several taxa were positively associated with AGP concentration including *Ruminococcus gnavus*, Lactobacillus and Clostridiales. Previous studies have reported an enrichment of Ruminococcus gnavus and Lactobacillus in children with IBD, which is consistent with our findings, while Clostridiales were depleted in individuals with IBD relative to healthy controls^9,39–41^, which is in conflict with our results. Several taxa present at 18 mo were positively associated with concurrent inflammation including Prevotella, *Clostridium innocuum* and Leuconostoc. While Prevotella may be beneficial because of its cellulolytic activities, it has also been linked to chronic inflammatory conditions such as arthritis as well as mucosal and systemic T-cell activation in HIV infected subjects not on therapy^42^. Because Prevotella is a genus comprised of many different species, it is possible that the differences in the observed metabolic functions attributed to Prevotella are due to different species present within the genus, thus the species diversity within a given genus ought to be taken into account when interpreting results.

Strengths of our study include a large sample size and a longitudinal approach to the analysis of the data, which allowed us to study the associations between microbiota characteristics and infant outcomes over time. In addition, our study included training of research personnel in good clinical practices, as well as maintaining a high level of standardization and quality assurance during data collection. One of the limitations was that there were some statistically significant differences in two baseline characteristics (number of food insecure households, household asset Z-score) between included and excluded participants. Although these may not necessarily affect the conclusions drawn from the analysis, they may affect the generalizability of the findings to the study population. Another limitation is the exploratory nature of this study. While we had pre-specified hypotheses for the associations of the outcomes with MAZ and Shannon index, the taxa-specific analyses were entirely exploratory and therefore hypothesis generating. We also conducted multiple hypothesis testing but did not perform a statistical correction for multiple hypothesis testing (except for the taxa-specific analyses) because the growth outcomes are closely related to each other. Thus, it is possible that some findings could be due to chance.

In conclusion, microbiota maturity and diversity (at 6 mo) were associated with growth in weight but not length between 6 and 12 mo, while no such association was observed between microbiota characteristics at 12 mo and growth outcomes between 12 and 18 mo, in our setting. These findings suggest that microbiota characteristics may play an important role in weight gain during the second half of the first year of life. Microbiota diversity and maturity may also be linked to reduced inflammation, but findings were inconsistent, and the potential causal direction is unclear. We recommend further research in other settings to evaluate whether these associations are replicated.

## Methods

### Study setting and design

The data for the study reported in this article were obtained from a clinical trial conducted in Malawi. The details of the study known as the International Lipid-based Nutrient Supplements DYAD (iLiNS-DYAD) trial have been reported previously^43,44^. Briefly, we enrolled 1391 pregnant mothers above 15 years of age and ≤20 gestational weeks from the antenatal clinics of two health centres and two hospitals in Mangochi district. Based on the sample size needed for the original iLiNS-DYAD trial, 869 mothers were allocated to 18 mo follow-up study after delivery and the singleton infants born to these mothers were participants for the current analysis. Infants born to the remaining 522 women who were assigned to pregnancy intervention only were not included (Fig. 1).

At baseline, the following data were collected by trained study personnel: Socio-demographic status, maternal age, height, body mass index (BMI), parity, education, HIV status, hemoglobin concentration, household assets, food security, source of drinking water (tap water vs any other source), access to sanitary facility (water closet or ventilation improved pit latrine vs. none or regular pit latrine) and season. We established maternal HIV status using a whole-blood antibody rapid test (Alere Determine HIV-1/2, Alere Medical Co, Ltd. and Uni-Gold HIV; Trinity Biotech plc)^44^. Details of the trial have been recorded at the clinical trial registry at the National Institutes of Health (USA) (www.clinicaltrials.gov), under the registration number NCT01239693. The trial was conducted by adhering to the Good Clinical Practice guide-lines and ethical standards of the Helsinki Declaration. We obtained ethical clearance for the study from the University of Malawi College of Medicine Research and Ethics Committee (COMREC) and the ethics committee at Tampere University Hospital District, Finland. An informed consent was obtained from each participant before being enrolled into the study. An independent data safety and monitoring board monitored the incidence of suspected serious adverse events (SAEs) during the trial.

### Faecal sample collection

Mothers were trained to collect faecal samples (generally in the morning) from participating children in their homes at 6, and 12 mo of age. The mothers were provided with sample collection tubes a day before the scheduled sample collection visit. Samples from suspected diarrhoea cases (>3 stools a day and markedly more liquid) were excluded, and the visit was rescheduled for two weeks later. The tubes containing faecal matter were sealed, labelled, and immediately stored in a Ziploc bag on a frozen ice pack in a cooler bag. The samples were transported to a satellite clinic within 6 hours of sample collection for a brief storage at −20 °C before being transported to the central clinic in Mangochi for storage at −80 °C within 48 hours. The samples were later shipped on dry ice to the USA for culture-independent analysis of community composition at Washington University, St. Louis, MO. Sample collection spanned all the three seasons of Malawi: warm-wet season (November-April), cool-dry-winter season (May-August), and hot-dry season (September–October).

### DNA Purification and 16S rRNA Sequencing

The isolation of DNA from stool and 16S rRNA gene amplicon sequencing was conducted as described elsewhere^45^. Stool samples were homogenized by grinding in the presence of liquid nitrogen prior to DNA extraction. DNA libraries were prepared by amplifying the V4 region (~255 bp) of the 16S rRNA gene. The DNA libraries were then sequenced on the Illumina MiSeq platform. Sequence processing and picking of clusters of closely related sequences (operational taxonomic units (OTUs) at 97% sequence identity) were performed in QIIME version 1.9.1^46^. OTU data were filtered using a threshold of at least 0.1% of sequence reads in two or more samples.

### Measurements of microbial maturity and diversity

We employed a Random Forests machine learning model to determine microbiota maturity^15,17^. The model was generated from an analysis of faecal samples collected from members of a Malawian cohort from birth through the second year of life. The model predicts microbiota age (state of development) based on the abundances of 25 age-discriminatory OTUs^17^. Microbiota ages of study members predicted by this model were compared to the median microbiota age of chronologically age-matched children in the healthy reference group to generate microbiota-for-age Z-scores (MAZ-scores). The data for the healthy reference group of children were obtained from healthy Malawian children and microbiota maturity was calculated as reported earlier^15^.

Microbiota diversity (based on mean alpha diversity of OTUs in each sample) was measured by calculating the Shannon diversity index using the phyloseq package in R^47^. Shannon index takes into account both richness and evenness of OTUs in each sample. A larger value indicates higher level of diversity^48^. For these analyses, Shannon index and MAZ scores were calculated at 6, 12 and 18 mo. Shannon index and MAZ scores were calculated with OTU data that were rarefied to 5000 reads.

### Measurement of growth and inflammation outcomes

Growth was assessed as described previously^43^. The z-score growth variables (WAZ, LAZ, WLZ, HCZ) were calculated by standardizing for age and sex using the WHO Child Growth Standards^49^. The changes in LAZ, WAZ, WLZ, and HCZ were calculated by taking the difference in z-score between time points, and then dividing by the number of days between the two measurements. The value was then multiplied by the standard number of days for each 6-mo period. Values below −2.0 for WAZ, LAZ, WLZ and HCZ were considered to indicate underweight, stunting, wasting and small head circumference, respectively.

To assess inflammation, Alpha-1 acid glycoprotein (AGP) and C-reactive protein (CRP) were analysed from plasma by immunoturbidimetry on the Cobas Integra 400 system auto-analyser (F. Hoffmann-La Roche Ltd, Basel, Switzerland) and reported as g/L and mg/L respectively. High AGP was defined as having AGP value >1.0 g/L and high CRP as having CRP value >5.0 mg/L.

### Statistical analysis

All data were analysed using SAS version 9.4 (Cary, NC). Children whose age at a given time point was outside of the pre-specified range for these analyses were excluded. At 6 mo children older than 8 mo were excluded (n = 13), at 12 mo children older than 15 mo were excluded (n = 5) and at 18 mo children older than 21 mo were excluded (n = 2). AGP and CRP were natural log transformed and the β-values and confidence intervals were back-transformed. For continuous outcomes, linear regression (proc glimmix) was used and the β-values and SEs of the predictor are presented. For dichotomous outcomes, logistic regression (proc glimmix) was used and the odds ratios and confidence intervals are presented. These models were fully adjusted for the following pre-specified covariates: intervention group; child age on day of stool collection; maternal age, height, body mass index, parity, education, HIV status, and hemoglobin at enrollment; household assets, food security, source of drinking water (tap water vs any other source), residential location and access to sanitary facility (water closet or ventilation improved pit latrine vs. none or regular pit latrine); season at time of stool sample collection; mode of delivery (vaginal or cesarean); site of delivery; and child sex. The residuals were assessed for outliers and normality. Extreme values were winsorized to the 2.5^th^ and 97.5^th^ percentile, and a sensitivity analysis was conducted with and without winsorized values. The models were inspected for multicollinearity and any covariate that was associated with the predictor with a Spearman’s correlation coefficient greater than 0.7 was removed.

To address the hypotheses regarding growth outcomes, we used repeated measures ANCOVA to assess whether Shannon index or MAZ were predictive of change in anthropometric z-scores. The model included two age intervals. For the first age interval, the response variable was the change in anthropometric z-score between 6 and 12 mo and the predictor was MAZ or Shannon index at 6 mo. The anthropometric z-score at 6 mo was included as a covariate to control for status at the beginning of the time interval. For the second age interval, the response variable was the change in anthropometric z-score between 12 and 18 mo, the predictor was MAZ or Shannon index at 12 mo, and the growth measurement z-score at 12 mo was included as a covariate. If an observation for growth or microbiota was missing at one time point, the observation was included for the other time point. The beta-value of MAZ or Shannon index across both intervals was of interest but could not be interpreted from a model that also included the interaction between interval and predictor. Therefore, a separate model was conducted that contained a term for the age interval and a term for the predictor, and the beta-value from this predictor was reported. For these models, we first examined the interaction between time interval and the predictor. If this interaction was significant, it meant that the relationship of the predictor to the outcome differed between the two age intervals, and we then assessed each age interval separately. The β-values of the predictor are presented. These models were fully adjusted and include the covariates previously described.

To test the hypothesis that MAZ and Shannon index are related to concurrent inflammation, we performed linear regression in which the predictor was either MAZ or Shannon index at 6 mo, and the outcome was inflammation as measured by log-transformed CRP or log-transformed AGP. Additionally, logistic regression models were performed to assess whether MAZ or Shannon index at 6 mo was associated with high AGP or high CRP. To test the hypothesis that MAZ and Shannon index are related to future inflammation, we repeated the models described above, except that the outcomes were measured at 18 mo and the predictors were measured at 6 or 12 mo. The β-values or odds ratios of the predictor are presented. These models were fully adjusted and include the covariates previously described.

For taxa-specific analyses, we used two filters to restrict the OTUs used in analysis. The first filter excluded OTUs that were present in less than 5% of the participants at a given time point. The second filter excluded OTUs with very low abundances of less than 0.1% in a given sample. To identify which taxa were related to growth and inflammation, we performed another set of regression model. We focused this analysis on outcomes for which there was a significant relationship with MAZ or Shannon index. The distributions of the OTU abundances were frequently zero-inflated and non-normal. Therefore, we condensed the OTU abundance data into two separate variables: a) a binary variable for whether the OTU was present (at any abundance), and b) a variable with three categories of OTU abundance (OTU absent, OTU present at a count less than the median, OTU present at a count greater than the median). These models were fully adjusted for all the pre-specified covariates. The Benjamini-Hochberg correction for multiple hypotheses was applied, using a false discovery rate (FDR) of 0.15.

## Data Availability

The datasets generated during and/or analysed during the current study are available from the corresponding author on reasonable request. The V4 16S rRNA sequence data generated in this study are available through the European Nucleotide Archive (Accession Number PRJEB29433).
